# Surface-Enhanced Impulsive Coherent Vibrational Spectroscopy

**DOI:** 10.1038/srep36471

**Published:** 2016-11-04

**Authors:** Juan Du, Juha Harra, Matti Virkki, Jyrki M. Mäkelä, Yuxin Leng, Martti Kauranen, Takayoshi Kobayashi

**Affiliations:** 1 State Key Laboratory of High Field Laser Physics, Shanghai Institute of Optics and Fine Mechanics, Chinese Academy of Sciences, Shanghai, 201800, China; 2 Department of Physics, Tampere University of Technology, P.O. Box 692, 33101, Tampere, Finland; 3 Ultrafast Laser Research Center, University of Electro-Communications, 1-5-1, Chofugaoka, Chofu, Tokyo, 182-8585, Japan; 4 JST, CREST, K’s Gobancho, 7 Gobancho, Chiyoda-ku, Tokyo, 102-0076, Japan; 5 Advanced Ultrafast Laser Center, National Chiao-Tung University, Hsinchu, 30010, Taiwan

## Abstract

Surface-enhanced Raman spectroscopy (SERS) has attracted a lot of attention in molecular sensing because of the remarkable ability of plasmonic metal nanostructures to enhance the weak Raman scattering process. On the other hand, coherent vibrational spectroscopy triggered by impulsive excitation using ultrafast laser pulses provides complete information about the temporal evolution of molecular vibrations, allowing dynamical processes in molecular systems to be followed in “real time”. Here, we combine these two concepts and demonstrate surface-enhanced impulsive vibrational spectroscopy. The vibrational modes of the ground and excited states of poly[2-methoxy-5-(2-ethylhexyloxy)−1,4-phenylenevinylene] (MEH-PPV), spin-coated on a substrate covered with monodisperse silver nanoparticles, are impulsively excited with a sub-10 fs pump pulse and characterized with a delayed broad-band probe pulse. The maximum enhancement in the spectrally and temporally resolved vibrational signatures averaged over the whole sample is about 4.6, while the real-time information about the instantaneous vibrational amplitude together with the initial vibrational phase is preserved. The phase is essential to determine the vibrational contributions from the ground and excited states.

Ever since its discovery^1^ in 1974 and interpretation^2^,^3^ in 1977, surface-enhanced Raman scattering (SERS) has proven to be an attractive technique for molecular sensing because it can remarkably enhance the weak Raman scattering process^4^,^5^,^6^,^7^,^8^,^9^. SERS is attracting a lot of attention, both in studies where deeper understanding of its details is expected to further boost the enhancement factor (EF) and in its application to biological systems. There are two different enhancement mechanisms that are usually considered to play a role in SERS, the electromagnetic mechanism and the chemical mechanism^10^,^11^. The former is directly associated with the strong electromagnetic fields that arise from the plasmonic excitations of nanostructured metals, e.g., rough surfaces or nanoparticles (NP)^12^,^13^. This mechanism is considered to be the dominant contribution to the dramatic increase of the Raman scattering cross section. The latter mechanism arises from a number of possible interactions between the molecule and the metal that modify the physico-chemical properties of the molecule, e.g., through a small charge transfer from the metal to the molecule, electron-photon coupling, and temporal movement of the adsorbed molecules^10^,^11^,^14^,^15^,^16^.

In spite of the great signal enhancement provided by SERS, nanoscale molecular sensing also requires short detection time, high temporal and spectral resolution, and low background^17^. Therefore, several vibrational laser spectroscopies, especially coherent anti-Stokes Raman scattering (CARS), have been combined with surface enhancement^17^,^18^,^19^,^20^,^21^,^22^,^23^. CARS spectroscopy, however, suffers from the non-resonant background signal, which is also enhanced by the plasmonic nanoparticles^17^,^21^,^22^,^23^. Other requirements for optimized CARS signals include strict selection of incoming beam frequencies and the phase matching condition. To suppress the non-resonant background, a new time-resolved surfaced-enhanced CARS technique employing femtosecond pump and Stokes pulses to excite molecular vibrational coherence and a picosecond narrowband probe pulse to generate the CARS signal has been developed^17^. However, in such experiments, the frequency of the vibrational signal is limited by the spectral separation between the femtosecond pump and Stokes pulses arriving at the sample simultaneously.

In addition, most SERS efforts have been focused on the vibrations of the electronic ground state of the molecules, which is a limitation of conventional Raman spectroscopy as such. It is evident that the vibrations of the excited states can provide complementary information about the molecular system.They play a crucial role in the ultrafast structural dynamics of photochemical reactions of the molecules^18^,^24^, including proton transfer in green fluorescent protein^25^ and state changes during ultrafast intersystem crossing in the dye tris(bipyridine)ruthenium(II) (Ru(bpy)_3_^2+^)^26^. Surface-enhanced excited-state Raman scattering (SEERS) was first reported in 1986, showing that enhancement is also applicable to the vibrations of the excited state^27^. Here, the excited-state vibrations were accessed by first pumping the molecule to the excited state and using this state as the initial state of the Raman process. More recently, other Raman processes, for example femtosecond stimulated Raman spectroscopy (FSRS) and CARS have been combined with surface enhancement. For a review of such processes, see, e.g., ref. 18. However, all these spontaneous, stimulated, and coherent Raman processes require a pump pulse to excite the molecules before the probe and Raman pulses interact with them. This makes the laser system very complex and it becomes difficult to maintain its stability during the measurements.

Due to the complementary character of the information provided by the ground- and excited-state vibrations, it is desirable to address them simultaneously to provide full information about the molecular dynamical processes. This can be achieved by resonant Raman techniques, such as surface-enhanced resonant Raman scattering (SERRS)^28^ or coherent anti-Stokes resonant Raman scattering (CARRS)^29^,^30^,^31^. However, in order to provide the ground and excited state information at the same time, the ratio between the ground and excited state vibrations needs to be controlled by adjusting the pump intensity during the measurements. This is not easy to do without a priori information about the absorption saturation levels and Raman scattering cross sections.

The ground and excited state information can be accessed simultaneously also by impulsive vibrational spectroscopy, which has already proven to be a powerful method to investigate condensed matter, including molecules, polymers, and biopolymers^24^,^32^,^33^,^34^,^35^. The technique is based on using an ultrashort pump laser pulse to excite a coherent wave packet of vibrational molecular motion, subsequently probed by a delayed probe pulse. In order to do this, the pump pulse must be much shorter than the period of molecular vibrations. The wave packet can be generated by impulsive excitation of several vibrational levels in the excited state (split excited state, V-type excitation) and/or by impulsive stimulated Raman scattering in the ground state (split ground state, Λ-type excitation), both pathways well-known from coherent laser spectroscopy^36^. The coherent molecular motions in the vibrational manifolds of the electronic ground and excited states give rise to temporal modulation in the transition probability between these states, which can be spectrally resolved from the time-dependent absorption of the probe pulse. The time delay between the degenerate probe and pump pulses can be precisely determined, allowing the temporal evolution of the vibrational modes (both amplitude and phase) to be followed. Although the technique relies on repetitive signals, it provides such a complete picture of the dynamical processes in molecular systems that it is often referred to as a “real-time” spectroscopic technique.

In this Paper, we combine the advantages of surface enhancement and impulsive laser spectroscopy to simultaneously address both the ground- and excited-state vibrations in the time scale of molecular motions. This technique fully satisfies the requirements for nanoscale real-time molecular sensing, which include small background, rapid signal acquisition, high enhancement, good reproducibility, as well as high temporal and spectral resolution. This possibility is demonstrated by using thin films of a polymeric semiconductor, poly[2-methoxy-5-(2-ethylhexyloxy)−1,4-phenylenevinylene] (MEH-PPV), spin-coated on a substrate covered with monodisperse silver (Ag) NPs. Superpositions of vibrational eigenstates of the electronic ground and excited states with several different quantum numbers are triggered by the impulsive excitation with sub-10 fs laser pulses^37^, which give rise to coherent wave packet motions in the molecules. The local-field enhancement supported by the Ag NPs is shown to couple to the dynamics of molecular vibrations associated with the electronic transition to the lowest excited state of MEH-PPV. The vibrational modes due to both excited- and ground-state wave packet motions are excited and the EF is recorded as a function of the probe wavelength. We find that the time-dependent spectroscopic signals, averaged over the whole sample volume, can be enhanced by a factor of 4.6 compared to a similar reference sample with no NPs. Importantly, the temporal shape of the signals remains essentially unchanged in spite of the enhanced signal levels.

## Results

### Sample characterization

**Figure 1:**
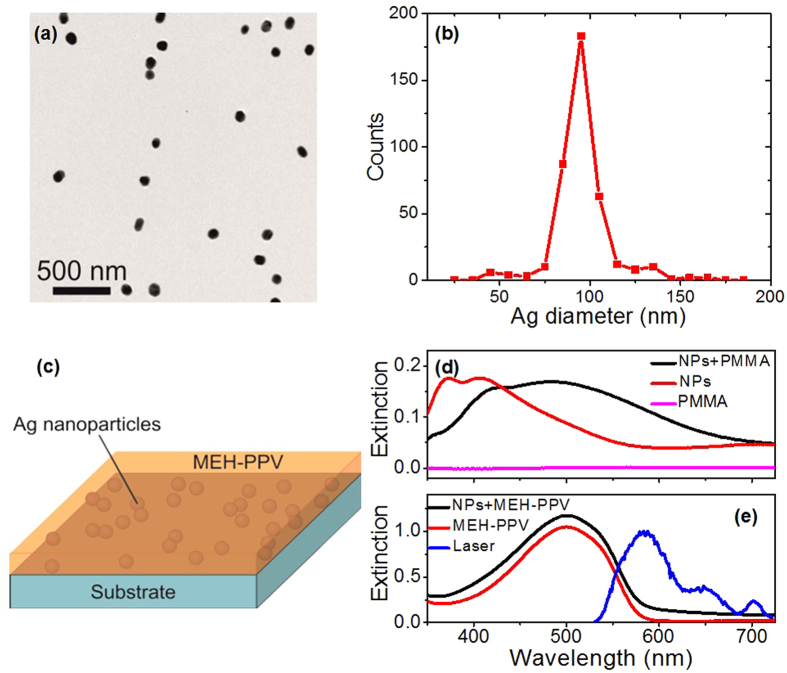
Sample characteristics. **(a)** TEM image of Ag NPs fabricated by aerosol techniques; **(b)** NP size distribution; **(c)** Schematics of the prepared sample; **(d)** Extinction spectra of Ag NPs (red line), PMMA (purple line), and NPs coated with PMMA (black line); **(e)** Extinction spectra of the MEH-PPV film as reference (red line), NP/MEH-PPV sample (black line), and the laser spectrum (blue line).

Our samples were thin films of MEH-PPV spin-coated on top of spherical and monodisperse Ag NPs supported by a glass substrate. Figure 1 shows a transmission electron microscope (TEM) image of the synthesized Ag NPs (Fig. 1(a)) along with the particle size distribution calculated from the micrographs (Fig. 1(b)). The thickness of the polymer layer was approximately 140 nm. An illustration of a prepared sample is shown in Fig. 1(c). Our reference sample was an identically prepared film of MEH-PPV without the NPs. A sample with NPs coated with poly(methyl methacrylate) (PMMA) was fabricated in order to study the absorption of the NPs in an environment similar to MEH-PPV but with no absorption from the polymer.

The extinction spectra of the samples are shown in Fig. 1(d). The spectrum of the NPs peaks in the 350–450 nm wavelength range. Importantly, the surface coverage of the NPs is sufficiently low that their plasmonic spectra are not significantly affected by aggregation^38^. However, when covered with the PMMA layer (Fig. 1(d)), which has no significant absorption above 300 nm wavelength, the spectrum is red-shifted and broadened to 350–700 nm range, in agreement with theoretical calculations^38^.

The ultra-fast pump-probe experiments were performed with a non-collinear optical parametric amplifier (NOPA) seeded by a white-light continuum^32^,^33^,^37^. Its sub-10 fs laser pulse covers the spectral range of 540–743 nm (Fig. 1(e)), therefore it interacts with the longer wavelength part of the plasmon resonance. In contrast to studies of conventional SERS, where the laser needs not be resonant with the molecular transition, enhancement of impulsive spectroscopy requires an overlap between the spectra of the laser, the metal NPs, and the electronic transition of the molecules. Figure 1(e) shows that this condition is achieved in our experiments at the long-wavelength part of the MEH-PPV spectrum. We are therefore exciting the lowest excited state of MEH-PPV. Note also that the spectrum of the NP/MEH-PPV sample is dominated by MEH-PPV with the NPs making a more significant relative contribution only at the very red end of the spectrum.

### Pump-probe spectroscopy

**Figure 2:**
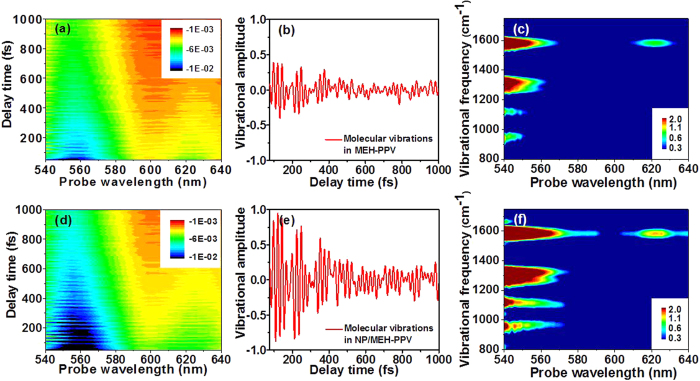
Spectral results with/without NPs. Show in **(a,b,c)** are experimental results for MEH-PPV, and shown in **(d,e,f)** are those for NP/MEH-PPV. **(a,d)** Two-dimensional (probe wavelength versus probe delay time) difference absorption spectra (obtained by taking difference between the probe absorption spectra with and without the pump laser); **(b,e)** oscillating components at the 556 nm probe wavelength, extracted from **(a,d)**, respectively; **(c,f)** two-dimensional contour plots of molecular vibrational intensity spectra obtained by taking Fourier transforms of **(a,d)**, respectively, and plotted using logarithmic scales.

The results for pump-probe spectroscopy are shown in Fig. 2(a–c) for the MEH-PPV reference sample and in Fig. 2(d–f) for the NP/MEH-PPV sample. It is clear from even superficial comparison of the two cases that the NPs do enhance the signals. The time-dependent changes in the absorption for different probe wavelengths are shown in Fig. 2(a,d), respectively. Note that the spectral lines are essentially “horizontal” as a function of time, which provides evidence that the measurements are not affected by any appreciable chirp of the laser spectrum. This is important because any chirp would necessitate more advanced data analysis^39^. The oscillating components, which are due to molecular vibrations, are then obtained from the time-dependent spectra. They are shown in Fig. 2(b,e) for the probe wavelength of 556 nm. Note that, in order to gain well-separated vibrational signatures from the experimental time traces, we have subtracted from the traces the components associated with slow-dynamics decay of the signals. Comparison of Fig. 2(b–e) shows directly that the molecular vibrations are enhanced by the NPs.

After acquiring the time-resolved absorbance changes, Fast Fourier transform (FFT) yields the vibrational mode frequencies and their respective intensities for different probe wavelengths, as shown in Fig. 2(c,f). The vibrational peaks at 969, 1112, 1262, 1319, and 1580 cm^−1^ are attributed to the out-of-plane CH bending mode of the vinylene group, mixtures of C-C stretching and C-H stretching band of benzene ring, ring deformation, vinyl C-H in-plane bending, vinyl C = C stretching and ring stretching, respectively^40^, which also appear in the Raman spectrum of MEH-PPV. All the vibrational modes of the MEH-PPV reference sample appear also in the spectra of the NP/MEH-PPV sample, but the latter are much stronger due to the enhancement provided by NPs.

**Figure 3:**
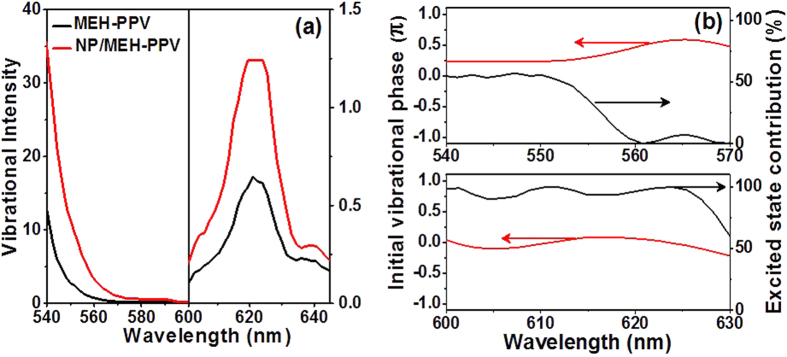
Enhancement of molecular vibrations due to NPs. **(a)** Probe wavelength dependence of the vibrational amplitude for the 1580 cm^−1^ mode in the MEH-PPV reference sample (black line) and in the NP/MEH-PPV film (red line). Note the different vertical scales for the left and right parts of the graph; **(b)** Initial vibrational phase and excited state wave-packet motion contribution to the 1580 cm^−1^ mode.

Since both the pump and probe spectra cover a broad bandwidth, our technique is capable of resolving complex probe-wavelength-dependent features associated with the vibrational modes (Fig. 2), which could not be resolved by conventional SERS. The amplitude of the strongest vibrational mode at 1580 cm^−1^ is plotted as a function of the probe wavelength in Fig. 3(a) for the MEH-PPV reference sample and the NP/MEH-PPV sample. Note in particular the wavelength band near the 620 nm probe wavelength, which is beyond the stationary absorption range of MEH-PPV peaking at ~500 nm (Fig. 1(e)). The main emission band of MEH-PPV is at about 565 nm^41^, which differs from 620 nm roughly by the vibrational frequency of 1580 cm^−1^. The band at 620 nm therefore corresponds to coherent macroscopic polarization, arising from energy exchange between molecular vibration at 1580 cm^−1^ and the field at ~565 nm, i.e., laser-Stokes energy exchange^42^,^43^. However, stimulated emission can also contribute to this band after vibrational dephasing time, occurring from the excited state with zero vibrational quanta to the ground state with one vibrational quantum (0−1 emission)^41^. For the present paper, the most important result is that the shapes of the traces in Fig. 3(a) at the key probe wavelength bands of about 540–560 nm and 600–630 nm are quite similar, indicating that the Ag NPs do not affect the spectra of the vibrational amplitude distribution but only enhance their strength. The results for the other vibrational modes are similar.

The information about the initial vibrational phase allows the vibrations of the ground and excited electronic states to be separated from each other^44^,^45^. The initial vibrational phase denotes the position where the coherent wave packet motion along the potential energy surface starts for each probe wavelength, which can be extracted from the pump-probe signal by the linear prediction singular value decomposition (LP-SVD) method^45^. If the initial vibrational phase *φ* is π/2 or 3π/2 (0 or π), the vibrational frequency is due to the wave-packet motion in the ground (excited) state^44^,^45^,^46^. The vibrational phases for all the observed modes are quite complicated. For example, for the strongest mode at 1580 cm^−1^, the phase is neither π/2 (or 3π/2) nor 0 (or π) in the range of 540–570 nm, but is nearly constant 0 in the range of 600–630 nm, as shown in Fig. 3(b). Therefore, the former has contributions from both the ground-state and the excited-state wave packets, while the latter has contributions mainly from the excited wave packet. The contribution from the excited-state wave packet is calculated by^44^,^45^,^46^ (cos *φ*)^2^, as shown in Fig. 3(b). Most important for the present work, however, is that the vibrational phases of MEH-PPV do not change after introducing the NPs, which suggests that the relative contributions to the vibrational signals from the ground and excited-state wave packets remain unchanged.

### Experimental and theoretical enhancement factor

**Figure 4:**
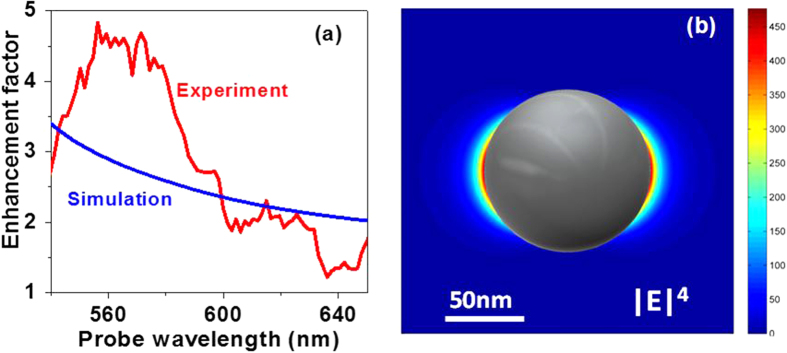
Experimental EF and simulation results. **(a**) Spectral distribution of the experimental EF (red line, calculated by a direct comparison of the data shown in Fig. 3) and the simulated EF (blue line); **(b)** The fourth power of the magnitude of the local electric field at 567 nm wavelength around a single Ag NP.

The EF in SERS is calculated by direct comparison of the intensities obtained by the SERS and Raman experiments, and normalized by the total number of molecules addressed in each experiment. However, in the present study, the density of NPs is quite low. Therefore, an approximate EF is obtained by dividing the vibrational intensity for the NP/MEH-PPV sample by that for the MEH-PPV reference. The EF spectra exhibit a much slower dependence on the probe wavelength (Fig. 4(a)), as expected on the basis of the spectral dependence of the plasmon resonance of the NPs. The peak EFs at these two bands are found to be 4.6 ± 0.2 and 2.1 ± 0.1, respectively. Other modes also exhibit similar EF spectra in the spectral range from 540 to 600 nm (not shown here). But only the 1580 cm^−1^ mode shows a clear band around 620 nm, so we select it as the example for our discussion.

The signal amplitude for pump-probe spectroscopy scales quadratically with respect to both pump and probe amplitudes. However, the reference spectrum is recorded independently for the MEH-PPV and MEH-PPV/NP samples. The probe enhancement therefore plays a role also in the reference measurement. The expected enhancement for the signal amplitude scales therefore quadratically with the pump amplitude and the signal intensity with the fourth power of the pump amplitude. However, this basic scaling is modified by the spectral details of the MEH-PPV ground-to-excited-state transition and the Ag NP plasmon resonances. In the following, we will neglect these spectral details to make an order-of-magnitude estimate for the expected enhancement in our sample.

We first calculate the absolute value of the local electric field for linearly polarized incident light near the Ag NPs embedded in a host with properties similar to MEH-PPV. For Ag, we use refractive index data from^47^ and for MEH-PPV from^48^ to deduce these quantities over the wavelength range of the experiments. Using standard approaches, the maximum field enhancement is found to be 4.65 at the NP surface for 567 nm which is at the center of the strongest enhancement observed experimentally (Fig. 4(a)). The maximum enhancement is not very high because our laser interacts only with the red end of the NP spectrum. In addition, the enhancement is not uniform even at the NP surface and it decays rapidly with increasing distance from the surface. Furthermore, the 8% surface coverage of NPs is relatively low. All of this reduces the amount of MEH-PPV experiencing the strongest enhancement. We have taken all of this into account in order to estimate the average enhancement that we can expect in the experiment.

The 8% surface coverage of 90 nm NPs embedded in a 140 nm MEH-PPV film implies that, on the average, each NP occupies a volume of 282 × 282 × 140 nm^3^. For simplicity, we consider that each NP is located at the center of a 282 × 282 × 140 nm^3^-sized unit cell touching one of the large facets. The local-field enhancement is close to unity at the small facets of the cell, which justifies neglecting the effect of other NPs when considering a single unit cell. We have therefore calculated the total field inside one unit cell with 1 nm grid spacing excluding the NP volume and integrated the fourth power (Fig. 4(b)) of the results over all three dimensions. The reference case is unity integrated over similar-sized unit cell to represent the signal from a sample without NPs. The result over the wavelength range of the experiments is shown in Fig. 4(a). The agreement with the measurements is reasonable although the spectral details of MEH-PPV are not accounted for. It is evident, however, that more advanced theoretical work will be necessary to explain the detailed experimental results. Such work is beyond the scope of the present paper, where the main goal was to experimentally demonstrate surface enhancement of impulsive ultrafast spectroscopy. Note also that our average EFs are comparable to the value of 13 observed for adenine on aluminum NP arrays in SERS experiments^8^. The reasonable agreement between experiment and theory allows us also to estimate the maximum enhancement of the vibrational intensity which is found at the NP surface. This quantity is (4.65)^4^ = 470 for the 567 nm wavelength. At this wavelength, the experimental enhancement from the whole sample exceeds the simulated one and this value can be considered as a lower estimate for the maximum enhancement.

### Time dependence of surface enhancement

**Figure 5:**
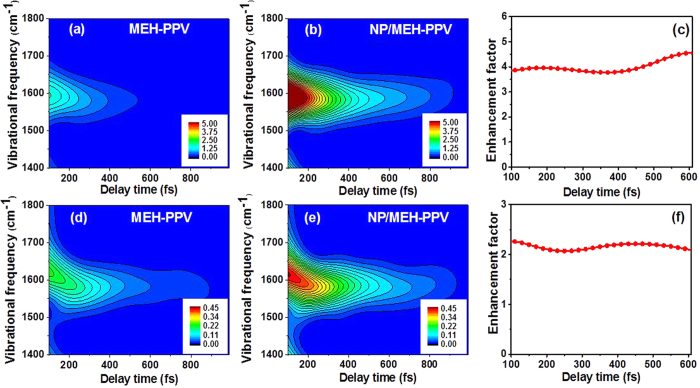
Time-evolution of the vibrational intensity. Shown in **(a,b)** are the time dependence of molecular vibrational intensity of the 1580 cm^−1^ mode probed at 556 nm for the MEH-PPV reference and the NP/MEH-PPV sample, respectively. **(c)** denotes the time-dependent EF at 556 nm. Shown in **(d,e,f)** are the corresponding results of the 1580 cm^−1^ mode probed at 620 nm.

In order to properly justify the use of the surface enhancement in real-time ultrafast spectroscopy, it is essential to investigate the behavior of the EF as a function of the pump-probe delay. The time-dependent vibrational information can be extracted from the measured data by the standard technique where the delay time is swept over the data and then Fourier transformed^49^. In more detail, we used a Blackman window function with FWHM width of 160 fs for sweeping. The time-dependent vibrational intensities of the 1580 cm^−1^ mode probed at 556 nm and 620 nm are shown in Fig. 5(a,b) and Fig. 5(d,e), respectively, for the MEH-PPV reference and the NP/MEH-PPV sample. By integrating the intensities from 1550 to 1610 cm^−1^ in the two graphs and comparing them, we obtain an average time-dependent EF at 556 nm (Fig. 5(c)) and 620 nm (Fig. 5(f)), respectively. It is clear that, in spite of the temporal decay of the intensities, the EF stays almost constant over the range of 100–600 fs for both bands. For even longer times, close to the longest measurement time, the reference signal becomes too noisy for a meaningful comparison. This result shows that the surface enhancement does not significantly compromise the quality of the time-dependent information that can be obtained from the sample. As mentioned before, the signal associated with the vibrational mode at 1580 cm^−1^ and probed at 556 nm is due to both the excited and ground states, and the one at 620 nm is due to the excited state only. The observed EFs in both ranges are nearly constant in time. It is therefore reasonable to conclude that both the excited and ground state vibrations undergo similar enhancements, which arises from the enhancement of the local fields that drive the process. This result is also in full agreement with the fact that the vibrational phases of the MEH-PPV reference and NP/MEH-PPV are identical as discussed in the context of Fig. 3.

## Discussion

We have provided direct evidence that impulsive coherent real-time vibrational spectroscopy can be enhanced by metal nanoparticles. In our specific experiments, we used Ag NPs to enhance the vibrational spectral information from the semiconducting polymer MEH-PPV. The enhancement factor was found to depend on the probe wavelength, with the highest value of 4.6 as averaged over the whole sample volume. In addition, the EF was shown to behave approximately as expected in relation to the spectral dependence of the plasmon resonance of the Ag NPs. Importantly, the EF was also shown to be essentially independent of the pump-probe delay time, showing that the time-dependent information is maintained by our technique.

Our experimentally determined enhancement factor averaged over the whole sample volume is in reasonable agreement with a theoretical estimate and suggests that the highest enhancement at the surface of the Ag nanoparticles is about 470. However, in order to approach this number in experiments, the number of molecules experiencing the highest enhancement would have to be significantly increased. The most obvious approach would be to bring the molecules only to the locations of the highest fields, but this may be experimentally challenging. Another possibility would be to increase the density of the NPs in the sample. Unfortunately, this may increase the probability of NP aggregation, which as such can either boost or suppress the achievable enhancement, depending on whether favorable oligomers with nanogaps are formed^9^. In addition, strong SERS from the aggregated metal colloids may hinder the signal from the molecules under study^10^. Further enhancement could also be expected from better matching of the laser and plasmon spectra. In the present work, we excited only the red tail of the plasmon resonance. On the other hand, it has been reported that the best excitation wavelength in SERS is a little blue-shifted with respect to the local surface plasmon resonance (LSPR) maximum^50^.

Of course, the general principle of surface-enhanced real-time spectroscopy is not limited to the material system studied in the present paper but could be extended to a number of other condensed-matter systems where ultrafast molecular motions play a central role. Unlike conventional SERS, where only the molecular ground state plays a role, or more complicated techniques for accessing the excited states, our method allows vibrational modes of both the ground and excited states to be investigated using only a single ultrafast laser source.

We expect that even higher enhancement factors could be achieved in optimized metal nanostructures, e.g., in those that rely on the field enhancement at sharp metal tips^51^. Additional opportunities are also expected to arise from approaches where SERS and pump-probe techniques are combined in innovative ways, as recently demonstrated^52^,^53^. Our surface-enhanced real-time vibrational spectroscopy could thus have potential applications in plasmon-driven chemistry, because the plasmon-generated hot electrons and plasmon-driven molecular dynamics could be monitored simultaneously due to the capability of detecting the instantaneous vibrational amplitude. Surface-enhanced ultrafast spectroscopies are therefore promising analytical techniques for ultrasensitive detection of dynamical processes in chemical and biomolecular systems. Such techniques can be used to increase the sensitivity of measurements, allowing the optical powers to be reduced which is essential for avoiding optical damage to fragile samples.

## Methods

### Experimental setup

Both the pump and probe pulses used in the present study were obtained from a non-collinear optical parametric amplifier (NOPA) seeded by a white-light continuum^37^. The pump source of the NOPA was a commercial regenerative amplifier (Spectra Physics, Spitfire), whose central wavelength, pulse duration, repetition rate, and average output power are 800 nm, 50 fs, 5 kHz, and 650 mW, respectively. The pulse duration of the NOPA output was compressed by a pair of Brewster angle prisms together with chirp mirrors. A typical duration of the output pulse was 6.8 fs with the spectral range extending from 540 nm to 743 nm. The energies of the pump and probe pulses were typically about 50 nJ and 6 nJ, respectively, which are low enough to avoid optical damage of NPs^17^. The resulting fs pulses were carefully characterized to be free from any appreciable chirp, which is important for reliable data analysis. The pump-probe signal was detected by a combination of a polychromator, a 128-channel lock-in amplifier and fiber bundles. The spectral resolution of the total system was about 1.5 nm. The wavelength-dependent absorbance changes were measured for pump-probe delay times from −200 fs to 1000 fs with a delay time step of 1 fs. All the experiments were performed at constant temperature (293 ± 1 K).

### Sample preparation

The NPs were synthesized in gas phase with aerosol techniques and deposited directly on the substrate, as described in earlier studies^38^,^54^. The diameter of the NPs was approximately 90 nm and the surface coverage of the prepared samples was estimated to be approximately 8%. MEH-PPV with Mn = 40,000–70,000 g/mol was acquired from Sigma-Aldrich and used without further purification. Thin films of the polymer were spin-coated from 1 wt.% solutions in chloroform on top of the substrates with the NPs.

### Theoretical model

The local electric field near metal nanoparticles embedded in a host medium was calculated using an electromagnetic approach. For a sphere that is small compared to the wavelength of the electromagnetic field, the scalar potential outside the sphere is^55^


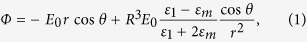


where *E*_0_ is the incident electric field amplitude, *r* is distance from the center of the sphere, *θ* is the angle between the position vector of the point in question and the incident field, *R* is the sphere radius and ε_1_ and ε_m_ are the permittivities of the sphere and the surrounding material, respectively.

For the electric field, we take the negative gradient of the potential, *E* = −∇*Φ*. As our scalar potential does not depend on the azimuthal angle, we can divide the electric field to the radial component *Er* and the angular component *E*_*θ*_. Applying the gradient in spherical coordinates and simplifying the expressions by using polarizability 
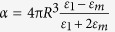
and a parameter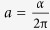, we obtain


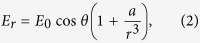



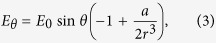


From these components we get the absolute total field near the NP


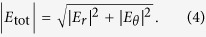


## Additional Information

**How to cite this article**: Du, J. *et al*. Surface-Enhanced Impulsive Coherent Vibrational Spectroscopy. *Sci. Rep.* **6**, 36471; doi: 10.1038/srep36471 (2016).

**Publisher’s note:** Springer Nature remains neutral with regard to jurisdictional claims in published maps and institutional affiliations.
